# Association of major and minor ECG abnormalities with traditional cardiovascular risk factors in the general population: a large scale study

**DOI:** 10.1038/s41598-024-62142-8

**Published:** 2024-05-17

**Authors:** Toktam Sahranavard, Rasoul Alimi, Javad Arabkhazaei, Mohamad Nasrabadi, Seyyed Mohammad Matin Alavi Dana, Yazdan Gholami, Azadeh Izadi-Moud, Habiobollah Esmaily, Mahmoud Ebrahimi, Gordon A. Ferns, Mohsen Moohebati, Sara Saffar Soflaei, Majid Ghayour Mobarhan

**Affiliations:** 1 International UNESCO Center for Health-Related Basic Sciences and Human Nutrition, Mashhad University of Medical Sciences, Mashhad, 99199-91766 Iran; 2 Department of Epidemiology and Biostatistics, School of Health, Torbat Heydariyeh University of Medical Sciences, Torbat Heydariyeh, Iran; 3 Faculty of Medicine, Mashhad University of Medical Sciences, Mashhad, Iran; 4 Department of Cardiovascular Diseases, Faculty of Medicine, Mashhad University of Medical Sciences, Mashhad, Iran; 5 Department of Biostatistics, School of Health, Mashhad University of Medical Sciences, Mashhad, Iran; 6 Vascular and Endovascular Research Center, Faculty of Medicine, Mashhad University of Medical Sciences, Mashhad, Iran; 7 Division of Medical Education, Brighton & Sussex Medical School, Falmer, Brighton, Sussex UK; 8 Heart and Vascular Research Center, Mashhad University of Medical Sciences, Mashhad, Iran

**Keywords:** Cardiology, Medical research

## Abstract

Cardiovascular disease (CVD) can be determined and quantified using the electrocardiogram (ECG) analysis. Identification of the risk factors associated with ECG abnormalities may advise prevention approaches to decrease CVD burden. In this study we aimed to investigate the association between CVD risk factors and minor and major ECG abnormalities in a general Iranian adult population. This study was conducted in 2010 and covered a population of 9035 males and females aged 35 to 65 years recruiting from the phase I of Mashhad Stroke and Heart Atherosclerotic Disorder (MASHAD) cohort study. The participants were drawn by a stratified cluster random sampling technique. The Bivariate and multinomial logistic regression analysis were conducted considering gender stratification to explore the association of ECG abnormalities with traditional cardiovascular risk factors. There was a significant association between minor and major ECG abnormalities and hypertension (HTN), type 2 diabetes (T2DM), smoking, and physical activity (*p* < 0.005). There was a significant trend, in both genders, for increasing major abnormalities as the number of CVD risk factors increased. But, only in women, the minor abnormalities increase in frequency as the number of CVD risk factors increased. The results of multinomial logistic regression showed that men with HTN [ARRR = 1.25, 95% CI 0.99, 1.57] and T2DM [ARRR = 1.31, 95% CI 0.99, 1.74] had the highest likelihood to have major abnormalities, although these are not statistically significant. For women, those with HTN had the highest likelihood to have major [ARRR = 1.36, 95% CI 1.13, 1.63] and minor [ARRR = 1.35, 95% CI 1.15, 1.58] abnormalities. Also, women aged > 60 years were more likely to have major [ARRR = 2.01, 95% CI 1.49, 2.74] and minor [ARRR = 1.59, 95% CI 1.20, 2.10] abnormalities compared to women aged < 45 years. Age and HTN were significantly associated with major and minor ECG abnormalities in women, and, on the other hand, HTN and T2DM were associated with major abnormalities in men. Taken together, these findings suggest that healthcare providers should advise preventive approaches to the asymptomatic adults with both major and minor electrocardiographic abnormalities that may predict cardiovascular risk.

## Introduction

Cardiovascular diseases (CVDs) represent the most widespread non-communicable diseases and hold the position of being the primary cause of mortality on a global scale^1^. According to a report by the World Health Organization, CVDs accounted for 32% of all recorded deaths^2^. The organization also reported an increasing trend in the mortality rate of CVDs, with an expected rise from 16.7 million deaths in 2010 to a forecasted 23.3 million deaths by the year 2030^3^. It is believed that over half of cardiovascular death can be attributed to the presence of following prominent risk factors: aging, hypercholesterolemia, hypertension (HTN), type 2 diabetes (T2DM), obesity, and smoking^4^. The prevalence of many risk factors of CVDs is increasing, particularly in developing regions^5^.

Despite the continuous progression of new technologies for the diagnostic evaluation of patients with CVD, the electrocardiogram (ECG) remains an essential tool^6^. Several studies have demonstrated promising prospects of utilizing the Minnesota ECG Code Classification System for the diagnosis of clinical types of coronary artery disease and heart failure, in addition to prognosticating CVD mortality^7–9^.

The ECG is a good candidate to use for risk stratification of apparently healthy participants due to its safety, wide availability, and low cost. Whereas routine ECG performance among healthy adults is not supported by present evidence^10^. If current CVD risk assessment tools could be enhanced, treatment might be improved, thereby maximizing the advantages of and minimizing the disadvantages of screening^11^.

Some studies reported the association of traditional CVD risk factors with ECG abnormalities^12,13^. However, it is not clear whether and to what extent these factors are associated with abnormal ECG changes in Iranian population. Identifying the groups at increased risk for ECG abnormalities may advise prevention approaches focused on modifiable risk factors to decrease the ECG abnormalities burden, which may in turn develop CVD prevention. This study was designed to explore the association of traditional CVD risk factors and major and minor ECG abnormalities on a large community in Iran.

## Methods

### Study population

This cross‐sectional study was carried out on the population of the first phase of Mashhad stroke and heart atherosclerotic disorder (MASHAD) cohort study. The MASHAD cohort study was initiated in 2010, consisting of 9704 participants aged 35 to 65 years. The population study were drawn from three regions in Mashhad by a stratified cluster random sampling technique. Details of the study design and sampling methods have been published previously^14^. Among the 9704 subjects, the ECGs of 9035 subjects were available for final analysis including 3615 (40.0%) males and 5420 (60.0%) females.

### Anthropometric assessments

Height, weight, and body mass index (BMI) were assessed regarding the standardized protocols^15^ in all subjects. Height (cm) was recorded to the nearest millimetre using a tape measure. Weight (kg) was measured to the nearest 0.1 kg using electronic scales. The BMI was evaluated by dividing weight (kg) to height squared (m^2^)^15^. Obesity was considered when BMI ≥ 30 kg/m^2^^16^.

### Laboratory evaluation

Blood samples of all subjects were gathered after a 14 h overnight fasting. Serum total cholesterol (TC) and fasting blood glucose (FBG) were estimated using enzymatic methods on an automated analyser. Hypercholesterolemia was defined as TC ≥ 240 mg/dl^17^. T2DM was described as FBG ≥ 126 mg/dl or previously diagnosed Type 2 T2DM^18^.

### Blood pressure assessment

Systolic blood pressure (SBP) and diastolic blood pressure (DBP) were measured by standard mercury sphygmomanometers. HTN was defined as a SBP ≥ 140 mmHg or a DBP ≥ 90 mmHg or a history of HTN^19^.

### Assessment of physical activity

The questionnaire utilized for evaluating physical activity was regarding the James and Schofield human energy requirements equations and was filled out by each participant. Questions were classified into three sections including the activities in work hours, off time, and in bed^20^. Individuals with 1–1.39 physical activity level were classified in inactive group and those who had PAL more than 1.4 were categorized as active ones^21^.

### Assessment of other variables

Demographic and socioeconomic features (e.g. age, gender, education, and occupation status) and smoking (current smoker, ever smoker and never smoker) for all subjects were recorded by health care professionals and a nurse interview.

### Electrocardiographic measures

A standard resting 12-lead ECG at a 25 mm/s paper speed and at 10 mm/mV was digitally recorded. All ECGs were inspected visually to detect technical errors, missing leads, and inadequate quality, and such records were rejected from electrocardiographic data files.

The readable ECGs (n = 9035) were subsequently examined and recorded by trained senior medical students regarding the Minnesota coding (MC) system^22,23^. ECG abnormalities were divided as major and minor abnormalities based on the standards of the MC for ECG classification^22^. Major ECG abnormalities were defined as any of the following: major Q wave abnormalities (MC 1-2, 1-2), Minor Q wave abnormalities Plus ST-T abnormalities (MC 4-1 or 4-2, or 5-1 or 5-2), Major isolated ST-T abnormalities (MC 4-1 or 4-2 or 5-1 or 5-2), complete or intermittent left bundle branch block (LBBB) (MC 7-1), right bundle branch block (RBBB) (MC 7-2), nonspecific intraventricular block (MC 7-4), RBBB with left anterior hemiblock (MC 7-8), Brugada pattern (MC 7-9), left ventricular hypertrophy plus ST-T abnormalities (MC 3-1 plus MC 4-1 or 4-2), Major QT index ≥ 116%, where QT Index = [QT interval × (heart rate + 100)/656]^24^, atrial fibrillation or flutter (MC 8-3), Third-degree AV conduction (AVB3) (MC 6-1), second-degree AV block (AVB2) (MC 6-2), WPW (MC 6-4), artificial pacemaker (MC 6-8), ventricular fibrillation or ventricular asystole (MC 8-2), supraventricular tachycardia (SVT) (MC 8-4-2 or MC 8-4-1 with Heart rate > 140). Minor abnormalities included: minor isolated Q/QS waves (MC 1-3), minor ST/T abnormalities (MC 4-3, 4-4, 5-3, 5-4), high R wave (left ventricular) (MC 3-1, 3-3, 3-4), high R wave (right ventricular) (MC 3-2), ST segment elevation (MC 9-2), incomplete RBBB (MC 7-3), incomplete LBBB (MC 7-6, 7-7), minor QT prolongation (QT index ≥ 112%), short PR interval (MC 6-5), long PR interval (MC 6-3), left axis deviation (MC 2-1), right axis deviation (MC 2-2), premature supraventricular beats (MC 8-1-1),premature ventricular beats (MC 8-1-2), premature combined beats (MC 8-1-3, 8-1-5), wandering atrial pacemaker (MC 8-1-4), sinus tachycardia (MC 8-7), sinus bradycardia (MC 8-8), supraventricular rhythm persistent (MC 8-4-1), low voltage QRS (MC 9-1), high amplitude P wave (MC 9-3), left atrial enlargement (LAE) (MC 9-6-), fragmented QRS (MC 7-1-0). Participants with both major and minor abnormalities were classified as having major abnormalities. Participants without minor or major ECG abnormalities were classified as having marginal or no abnormalities and their ECG was considered normal^10^.

### Statistical analysis

In this study, both descriptive, bivariate and multinomial logistic regression analysis were conducted considering gender stratification. The quantitative results were stated as mean and standard deviation and the qualitative results as frequency and percentage. Independent t-test and chi-square tests were used to compare the variables by sex. Bonferroni adjustments was used for multiple testing. Additionally, Multinomial logistic regression model was employed because the dependent variable had three outcomes (normal, minor and major). The results for the multinomial logistic regression analyses were presented as relative risk ratios (RRR) with their respective 95% confidence intervals (CIs) signifying precision. Also, to eliminate the effect of confounders, adjusted relative risk ratios (ARRR) along with their respective 95% confidence intervals (CIs) were used. All the analyses were done with SPSS version 26.

### Ethical approval and consent of participant

The Human Research Ethics Committee of Mashhad University of Medical Sciences (MUMS) reviewed and approved the study (IR.MUMS.MEDICAL.REC.1399.783). All subjects provided written informed consent. For illiterate participants, their literate spouse or children read and sign the form. All study procedures were conducted according to the World Medical Association Declaration of Helsinki ethical standards for medical research^25^.

## Results

Baseline characteristics of the participants by gender is showed in Table 1. Average (± SD) of the age was 48.91 ± 8.40 years in men and 47.60 ± 8.09 years in women, respectively.

**Table 1:** Comparison of baseline characteristics by gender.

Characteristics	Total population (n = 9035)		*P*-value	All
	Male n (%)	Female n (%)		
Age (year)	48.91 ± 8.40	47.60 ± 8.09	< 0.001	48.13 ± 8.24
< 45	1248 (34.5)^a^	2112 (39.0)^b^	< 0.001	3360 (37.2)
45–60	1902 (52.6)^a^	2797 (51.7)^a^		4699 (52.0)
> 60	464 (12.8)^a^	506 (9.3)^b^		970 (10.7)
Job status				
Unemployment	301 (8.3)^a^	4463 (82.4)^b^	< 0.001	4764 (52.8)
Employment	2648 (73.3)^a^	707 (13.1)^b^		3355 (37.1)
Retired	665 (18.4)^a^	247 (4.6)^b^		912 (10.1)
Educational attainment				
Illiterate	264 (7.3)^a^	891 (16.5)^b^	< 0.001	1155 (12.8)
Primary	1178 (32.7)^a^	2456 (45.6)^b^		3634 (40.4)
High school	1508 (41.8)^a^	1673 (31.0)^b^		3181 (35.4)
Diploma and above	655 (18.2)^a^	370 (6.9)^b^		1025 (11.4)
Smoking habit				
Never smoker	2096 (58.0)^a^	4122 (76.1)^b^	< 0.001	6218 (68.8)
Ever smoker	549 (15.2)^a^	342 (6.3)^b^		891 (9.9)
Current smoker	970 (26.8)^a^	956 (17.6)^b^		1926 (21.3)
HTN				
Yes	1053 (29.2)^a^	1745 (32.3)^b^	< 0.001	2798 (31.0)
No	2556 (70.8)	3664 (67.7)		6220 (69.0)
T2DM				
Yes	486 (13.6)	787 (14.7)	0.165	1273 (14.3)
No	3075 (86.4)	4567 (85.3)		7642 (85.7)
Hypercholesterolemia				
Yes	292 (8.1)	647 (12.0)	< 0.001	939 (10.4)
No	3300 (91.9)	4749 (88.0)		8049 (89.6)
Physical activity				
Inactive	1892 (52.4)^a^	268 (5.0)^b^	< 0.001	2160 (24.0)
Active	1718 (47.6)^a^	5137 (95.0)^b^		6855 (76.0)
Obesity				
Yes	631 (17.8)^a^	2096 (39.4)^b^	< 0.001	2727 (30.8)
No	2917 (82.2)^a^	3223 (60.6)^b^		6140 (69.2)
ECG abnormalities				
Normal	2189 (60.6)^a^	3615 (66.7)^a^	< 0.001	5804 (64.2)
Minor	945 (26.1)^a^	1050 (19.4)^b^		1995 (22.1)
Major	481 (13.3)^a^	755 (13.9)^b^		1236 (13.7)

HTN, hypertension; T2DM, type 2 diabetes.

Different lower-case letters indicate a significant difference in baseline characteristics by gender.

The relationship between abnormal ECG changes and cardiovascular risk factors is presented in Table 2. There was a considerable association between minor and major abnormalities and HTN, T2DM, smoking, and physical activity.

**Table 2:** Association between cardiovascular disease risk factors and ECG abnormalities.

Characteristics	Normal ECG (n = 5804)	Minor ECG abnormalities (n = 1995)	Major ECG abnormalities (n = 1236)	All (n = 9035)	*p*-value
HTN					
Yes	1642 (28.3)^a^	682 (34.3)^b^	474 (38.5)^c^	2798 (31.0)	< 0.001
No	4156 (71.7)	1307 (65.7)	757 (61.5)	6220 (69.0)	
T2DM					
Yes	769 (13.4)^a^	288 (14.6)^a, b^	216 (17.8)^b^	1273 (14.3)	0.001
No	4962 (86.6)	1680 (85.4)	1000 (82.2)	7642 (85.7)	
Obesity					
Yes	1765 (30.9)	568 (29.1)	394 (32.5)	2727 (30.8)	0.113
No	3938 (69.1)	1384 (70.9)	818 (67.5)	6140 (69.2)	
Hypercholesterolemia					
Yes	594 (10.3)	216 (10.9)	129 (10.5)	939 (10.4)	0.738
No	5186 (89.7)	1767 (89.1)	1096 (89.5)	8049 (89.6)	
Smoking					
Never smoker	4031 (69.5)^a^	1316 (66.0)^b^	871 (70.5)^a^	6218 (68.8)	0.006
Ever smoker	541 (9.3)	218 (10.9)	132 (10.7)	891 (9.9)	
Current smoker	1232 (21.2)^a,b^	461 (23.1)^b^	233 (18.9)^a^	1926 (21.3)	
Physical activity					
Active	4460 (77.0)^a^	1460 (73.3)^b^	935 (75.8)^a, b^	6855 (76.0)	0.004
Inactive	1330 (23.0)	531 (26.7)	299 (24.2)	2160 (24.0)	

HTN, hypertension; T2DM, type 2 diabetes.

Different lower-case letters indicate a significant difference in cardiovascular disease risk factors by ECG group.

The comparison of the major and minor ECG abnormalities by CVD risk status or disease in men and women is shown in Tables 3 and 4. There was a significant trend, in both men and women, for increasing major abnormalities as the number of CVD risk factors increased. But, only for women, minor abnormalities increased as the number of CVD risk factors increased. The results showed that men with more than 3 CVD risk factors were more likely [RRR = 1.39, 95% CI 1.01, 1.93] to have major abnormalities compared to those with no CVD risk factor. Also, women with 1 and 2 risk factor and more than 3 CVD risk factors were more likely [RRR = 1.21, 95% CI 1.01, 1.45] and [RRR = 1.41, 95% CI 1.10, 1.81] to have major abnormalities compared to those with no CVD risk factor, respectively. Minor abnormalities increased about 50 percent [RRR = 1.50, 95% CI 1.21, 1.85] for women with more than 3 CVD risk factors compared to those with no CVD risk factor.

**Table 3:** Prevalence of ECG abnormalities by cardiovascular disease risk factor status in men (n = 3615).

Minnesota code abnormalities	No. CVD risk factors (%) of participants			*P*-value for trend
	No (n = 587)	1 or 2 (n = 2261)	≥ 3 (n = 767)	
Minor abnormalities	145 (24.7)	603 (26.7)	197 (25.7)	0.759
Minor isolated Q/QS waves	25 (4.3)	126 (5.6)	54 (7.0)	0.027
Minor ST/T abnormalities	12 (2.0)	47 (2.1)	24 (3.1)	0.152
High R waves (left ventricular)	14 (2.4)	20 (0.9)	5 (0.7)	0.004
High R waves (right ventricular)	1 (0.2)	1 (0.0)	1 (0.1)	0.888
ST segment elevation	55 (9.4)	210 (9.3)	61 (8.0)	0.330
Incomplete RBBB	1 (0.2)	1 (0.0)	2 (0.3)	0.511
Incomplete LBBB	5 (0.9)	8 (0.4)	4 (0.5)	0.462
Minor QT prolongation	14 (2.4)	80 (3.6)	26 (3.4)	0.366
Short PR interval	0 (0.0)	1 (0.0)	0 (0.0)	0.935
Long PR interval	1 (0.2)	8 (0.4)	0 (0.0)	0.428
Left axis deviation	27 (4.6)	115 (5.1)	42 (5.5)	0.469
Right axis deviation	3 (0.5)	8 (0.4)	1 (0.1)	0.218
Premature beats (supra ventricular)	3 (0.5)	5 (0.2)	1 (0.1)	0.180
Premature beats (ventricular)	9 (1.5)	23 (1.0)	10 (1.3)	0.781
Premature beats (combined)	–	–	–	–
Wandering atrial pacemaker	–	–	–	–
Sinus tachycardia	7 (1.2)	21 (0.9)	10 (1.3)	0.767
Sinus bradycardia	29 (4.9)	106 (4.7)	40 (5.2)	0.771
Supraventricular rhythm persistent	0 (0.0)	1 (0.0)	0 (0.0)	0.935
Low voltage QRS	8 (1.4)	41 (1.8)	9 (1.2)	0.682
High amplitude P wave	1 (0.2)	14 (0.6)	0 (0.0)	0.459
LAE	16 (2.7)	63 (2.8)	26 (3.4)	0.439
Fragmented QRS	9 (1.5)	38 (1.7)	21 (2.70	0.084
Early repolarization	11 (1.9)	35 (1.5)	13 (1.7)	0.840
Major abnormalities	71 (12.1)	290 (12.8)	120 (15.6)	0.044
Major Q wave abnormalities (Old prevalent MI)	39 (6.6)	164 (7.3)	77 (10.0)	0.014
Minor Q wave abnormalities plus ST-T abnormalities (possible old MI)	3 (0.5)	5 (0.2)	7 (0.9)	0.168
Major Isolated ST-T abnormalities	18 (3.1)	63 (2.8)	36 (4.7)	0.061
Complete or intermittent LBBB	2 (0.3)	13 (0.6)	5 (0.7)	0.461
Complete or intermittent RBBB	2 (0.3)	8 (0.4)	5 (0.7)	0.339
Nonspecific intraventricular block	2 (0.3)	8 (0.4)	5 (0.7)	0.339
RBBB with left anterior hemiblock	0 (0.0)	0 (0.0)	1 (0.1)	0.119
Brugada pattern	4 (0.7)	14 (0.6)	1 (0.1)	0.137
Left ventricular hypertrophy plus ST-T abnormalities	25 (4.3)	77 (3.4)	40 (5.2)	0.266
Major QT prolongation	7 (1.2)	41 (1.8)	9 (1.2)	0.848
Atrial fibrillation or flutter	1 (0.2)	0 (0.0)	1 (0.1)	0.908
AVB2)	0 (0.0)	1 (0.0)	0 (0.0)	0.935
Ventricular fibrillation or ventricular asystole	1 (0.2)	0 (0.0)	1 (0.1)	0.908

CVD, cardiovascular disease; RBBB, right bundle branch block; LBBB, left bundle branch block; LAE, left atrial enlargement; MI, myocardial infarction; AVB2, second degree AV block.

**Table 4:** Prevalence of ECG abnormalities by cardiovascular disease risk factor status in women (n = 5420).

Minnesota code abnormalities	No. CVD risk factors (%) of participants			*P*-value for trend
	No (n = 1612)	1 or 2 (n = 3018)	≥ 3 (n = 790)	
Minor abnormalities	292 (18.1)	570 (18.9)	188 (23.8)	0.003
Minor isolated Q/QS waves	50 (3.1)	158 (5.2)	33 (4.2)	0.047
Minor ST/T abnormalities	66 (4.1)	108 (3.6)	42 (5.3)	0.348
High R waves (left ventricular)	5 (0.3)	18 (0.6)	6 (0.8)	0.121
High R waves (right ventricular)	3 (0.2)	3 (0.1)	2 (0.3)	0.907
ST segment elevation	39 (2.4)	57 (1.9)	14 (1.8)	0.216
Incomplete RBBB	3 (0.2)	1 (0.0)	0 (0.0)	0.065
Incomplete LBBB	3 (0.2)	7 (0.2)	4 (0.5)	0.197
Minor QT prolongation	61 (3.8)	140 (4.7)	46 (5.9)	0.022
Short PR interval	4 (0.2)	5(0.2)	2 (0.3)	0.877
Long PR interval	2 (0.1)	9 (0.3)	5 (0.6)	0.036
Left axis deviation	48 (3.0)	92 (3.0)	22 (2.8)	0.860
Right axis deviation	3 (0.2)	6 (0.2)	4 (0.5)	0.203
Premature beats (supra ventricular)	4 (0.2)	8 (0.3)	6 (0.8)	0.085
Premature beats (ventricular)	13 (0.8)	38 (1.3)	15 (1.9)	0.022
Premature beats (combined)	0 (0.0)	1 (0.0)	0 (0.0)	0.815
Wandering atrial pacemaker	1 (0.1)	0 (0.0)	0 (0.0)	0.191
Sinus tachycardia	23 (1.4)	50 (1.7)	29 (3.7)	0.001
Sinus bradycardia	21 (1.3)	47 (1.6)	10 (1.3)	0.884
Supraventricular rhythm persistent	2 (0.1)	1 (0.0)	0 (0.0)	0.169
Low voltage QRS	37 (2.3)	51 (1.7)	13 (1.6)	0.179
High amplitude P wave	6 (0.4)	13 (0.4)	3 (0.4)	0.912
LAE	15 (0.9)	60 (2.0)	27 (3.4)	< 0.001
Fragmented QRS	25 (1.6)	38 (1.3)	18 (2.3)	0.361
Early repolarization	11 (0.7)	21 (0.7)	6 (0.8)	0.848
Major abnormalities	200 (12.4)	434 (14.4)	121 (15.3)	0.032
Major Q wave abnormalities (Old prevalent MI)	85 (5.3)	179 (5.9)	52 (6.6)	0.182
Minor Q wave abnormalities plus ST-T abnormalities (possible old MI)	4 (0.2)	19 (0.6)	3 (0.4)	0.372
Major Isolated ST-T abnormalities	83 (5.1)	197 (6.5)	48 (6.1)	0.195
Complete or intermittent LBBB	6 (1.9)	14 (1.7)	7 (3.2)	0.129
Complete or intermittent RBBB	2 (0.1)	4 (0.1)	5 (0.6)	0.030
Nonspecific intraventricular block	2 (0.1)	4 (0.1)	5 (0.6)	0.030
RBBB with left anterior hemiblock	0 (0.0)	0 (0.0)	0 (0.0)	–
Brugada pattern	2 (0.1)	4 (0.1)	1 (0.1)	0.971
Left ventricular hypertrophy plus ST-T abnormalities	87 (5.4)	206 (6.8)	51 (6.5)	0.165
Major QT prolongation	27 (1.7)	57 (1.9)	17 (2.2)	0.400
Atrial fibrillation or flutter	2 (0.1)	1 (0.0)	0 (0.0)	0.169
AVB2	1 (0.1)	0 (0.0)	1 (0.1)	0.741
Ventricular fibrillation or ventricular asystole	1 (0.1)	0 (0.0)	0 (0.0)	0.191

CVD, cardiovascular disease; RBBB, right bundle branch block; LBBB, left bundle branch block; LAE, left atrial enlargement; MI, myocardial infarction; AVB2, second degree AV block.

Tables 5 and 6 present the results on the multinomial logistic regression analysis on ECG abnormalities among men and women.

**Table 5:** Predictor variables for ECG abnormalities among male.

Variable	Base outcome (Normal)		Base outcome (Normal)	
	Minor	Major	Minor	Major
	RRR (95% CI)	RRR (95% CI)	ARRR (95% CI)	ARRR (95% CI)
Age				
< 45	Ref	Ref	Ref	Ref
45–60	1.15(0.98–1.36)	1.45(1.15–1.82)**	1.09(0.81–1.47)	1.22(0.95–1.56)
> 60	1.18(0.92–1.52)	1.78(1.30–2.43)**	1.13(0.94–1.35)	1.28(0.88–1.87)
Job status				
Unemployment	Ref	Ref	Ref	Ref
Employment	1.02(0.83–1.25)	0.67(0.52–0.85)**	1.02(0.69–1.40)	0.76(0.58–1.01)
Retired	1.04(0.75–1.43)	0.81(0.54–1.19)	0.98(0.81–1.29)	0.79(0.51–1.23)
Educational attainment				
Illiterate	1.39(1.01–1.93)*	1.13(0.74–1.71)	1.29(0.91–1.84)	1.13(0.72–1.79)
Primary	1.13(0.90–1.41)	1.05(0.80–1.39)	1.11(0.87–1.41)	1.11(0.82–1.49)
High school	0.96(0.77–1.19)	0.73(0.55–0.96)*	0.95(0.76–1.19)	0.81(0.61–1.08)
Diploma and above	Ref	Ref	Ref	Ref
Smoking				
Never smoker	Ref	Ref	Ref	Ref
Ever smoker	0.90(0.75–1.07)	1.23(0.97–1.56)	0.95(0.79–1.14)	1.20(0.94–1.55)
Current smoker	0.94(0.74–1.20)	1.18(0.86–1.63)	0.94(0.73–1.21)	1.11(0.79–1.55)
HTN				
Yes	1.13(0.96–1.34)	1.45(1.18–1.79)**	1.12(0.94–1.35)	1.25(0.99–1.57)
No	Ref	Ref	Ref	Ref
T2DM				
Yes	0.97(0.77–1.21)	1.47(1.13–1.92)**	0.96(0.76–1.22)	1.31(0.99–1.74)
No	Ref	Ref	Ref	Ref
Hypercholesterolemia				
Yes	0.92(0.70–1.23)	0.92(0.64–1.33)	0.90(0.67–1.21)	0.83(0.56–1.21)
No	Ref	Ref	Ref	Ref
Physical activity				
Active	Ref	Ref	Ref	Ref
Inactive	0.94(0.80–1.09)	1.09(0.89–1.33)	0.97(0.81–1.15)	1.08(0.86–1.35)
Obesity				
Yes	0.87(0.71–1.07)	1.00(0.77–1.29)	0.87(0.70–1.08)	0.92(0.70–1.21)
No	Ref	Ref	Ref	Ref

HTN, hypertension; T2DM, type 2 diabetes.

* < 0.05, ** < 0.01.

**Table 6:** Predictor variables for ECG abnormalities among Female.

Variable	Base outcome (Normal)		Base outcome (Normal)	
	Minor	Major	Minor	Major
	RRR (95% CI)	RRR (95% CI)	ARRR (95% CI)	ARRR (95% CI)
Age				
< 45	Ref	Ref	Ref	Ref
45–60	1.38(1.18–1.60)**	1.53(1.29–1.82)**	1.24(1.05–1.47)*	1.34(1.10–1.63)**
> 60	1.99(1.57–2.53)**	2.53(1.94–3.29)**	1.59(1.20–2.10)**	2.01(1.49–2.74)**
Job status				
Unemployment	Ref	Ref	Ref	Ref
Employment	0.91(0.63–1.32)	0.75(0.49–1.16)	1.08(0.72–1.60)	0.82(0.51–1.29)
Retired	0.94(0.68–1.30)	0.96(0.66–1.39)	0.89(0.62–1.30)	0.93(0.61–1.40)
Educational attainment				
Illiterate	1.59(1.14–2.21)**	2.08(1.41–3.08)**	1.45(0.99–2.14)	1.55(0.98–2.46)
Primary	1.56(1.15–2.11)**	1.67(1.16–2.40)**	1.52(1.07–2.16)*	1.45(0.94–2.22)
High school	1.20(0.88–1.65)	1.42(0.98–2.08)	1.27(0.90–1.79)	1.36(0.89–2.06)
Diploma and above	Ref	Ref	Ref	Ref
Smoking habit				
Never smoker	Ref	Ref	Ref	Ref
Ever smoker	0.97(0.81–1.17)	1.10(0.89–1.36)	1.02(0.85–1.24)	1.16(0.93–1.45)
Current smoker	1.20(0.88–1.64)	1.46(1.03–2.07)*	1.13(0.82–1.55)	1.28(0.89–1.84)
HTN				
Yes	1.52(1.32–1.76)**	1.68(1.43–1.97)**	1.35(1.15–1.58)**	1.36(1.13–1.63)**
No	Ref	Ref	Ref	Ref
T2DM				
Yes	1.24(1.03–1.50)*	1.35(1.09–1.67)**	1.04(0.85–1.28)	1.10(0.88–1.38)
No	Ref	Ref	Ref	Ref
Hypercholesterolemia				
Yes	1.23(1.01–1.51)*	1.09(0.85–1.38)	1.06(0.85–1.31)	0.90(0.70–1.15)
No	Ref	Ref	Ref	Ref
Physical activity				
Active	Ref	Ref	Ref	Ref
Inactive	0.97(0.70–1.33)	0.95(0.66–1.37)	0.97(0.70–1.36)	0.92(0.65–1.35)
Obesity				
Yes	1.09(0.95–1.26)	1.13(0.96–1.33)	1.01(0.86–1.17)	1.04(0.87–1.23)
No	Ref	Ref	Ref	Ref

HTN, hypertension; T2DM, type 2 diabetes.

* < 0.05, ** < 0.01.

With no abnormalities (normal) as the base outcome, the results showed that men with HTN had the highest likelihood [by 25%] to have major abnormalities, although it is not statistically significant. Also, those with T2DM had the highest likelihood [by 31%] to have major abnormalities, although it is not statistically significant.

Women aged 45–60 and > 60 years were more likely [by 34%] and [About 2 times] to have higher risk of major abnormalities compared to those aged < 45, respectively.

Also, those with HTN had the highest likelihood [by 36%] to have major abnormalities.

Women aged 45–60 and > 60 years were more likely [by 24%] and [by 59%] to have higher risk of minor abnormalities compared to those aged < 45, respectively. Women illiterate and primary education were more likely [by 45%] and [by 52%] to have minor abnormalities compared to those with diploma an above, respectively. Also, those with HTN had the highest likelihood [by 35%] to have minor abnormalities.

**Figure 1:**
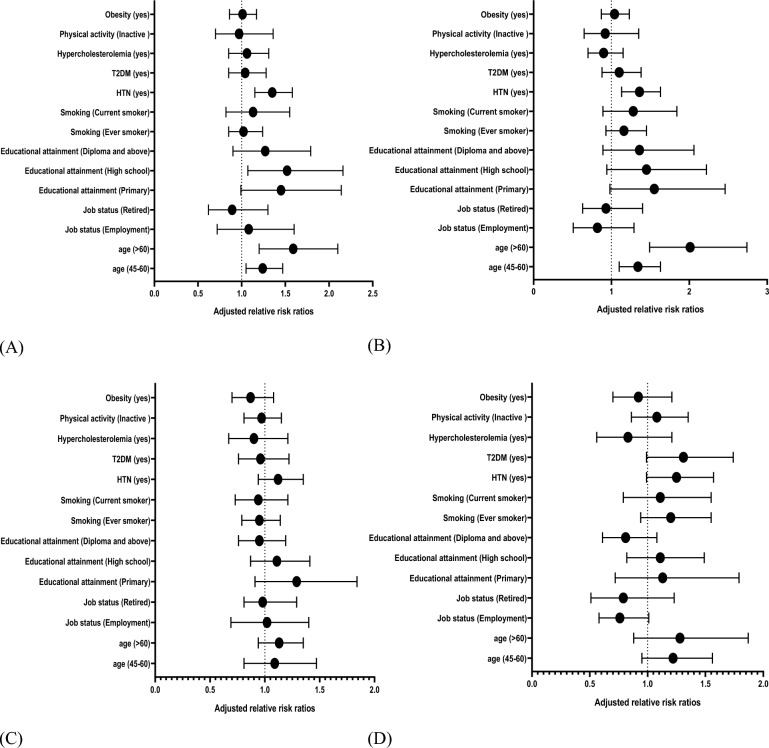
Adjusted relative risk ratios and confidence intervals of predictor variables for ECG abnormalities by gender. (**A**) Minor ECG abnormalities in female. (**B**) Major ECG abnormalities in female. (**C**) Minor ECG abnormalities in male. (**D**) Major ECG abnormalities in male.

The main findings of the Tables 5 and 6 are shown in Fig. 1. Figure 1 showed the adjusted relative risk ratios and confidence intervals for minor and major ECG abnormalities in male and female.

## Discussion

Our results showed that in a population-based study of men and women, minor and major ECG abnormalities were associated with age, history of HTN, and T2DM. An interesting finding of our study was that the major ECG abnormalities increased as the number of CVD risk factors increased in both men and women. But, only for women, minor abnormalities increased as the number of CVD risk factors increased.

After multinomial logistic regression analysis, only age and HTN remained significantly associated with major and minor ECG abnormalities in females. In males, age, HTN and T2DM were associated with major abnormalities. Although these associations were not statistically significant, they are clinically important.

The risk of CVD differs between men and women due to a protective impact of sex steroid hormones in women, particularly estrogen^26^. Therefore, we did all analysis in both genders separately.

This study showed that minor and major ECG abnormalities increased with aging in women. In line with our research, In both genders, the odds of presenting major ECG abnormalities surge with a rise in age^27,28^. The etiology of the observed ECG variations between age groups are not completely identified. These ECG differences between age groups may be contributed to developmental, electrical, and structural conduction system alternations in the heart throughout life^29^. Moreover, differences in the atherosclerosis and other CVDs risks, comorbidities, medications, and lifestyle parameters can also affect ECG components at different ages^28,30^.

HTN is one of the main risk factors for CVD. In our investigation, it was shown that HTN has a remarkable association with both minor and major ECG abnormalities in women. A similar study indicated that the ECG abnormalities were more commonly observed in the hypertensive group rather than the normotensive group^31^. In addition, a review study reported a significant association between HTN and ECG abnormalities including QTc prolongation and QRS wave fragmentation^32^. Our finding supports that evidence declaring that hypertensive condition can results in cardiac structure and electrical abnormalities^33^.

T2DM is another risk factor of CVD that is associated with major ECG abnormalities in men. In consistent with our results, Sahil et al. investigated the prevalence of ECG abnormalities in asymptomatic diabetic patients. Twenty-six percent of diabetic patients had ECG abnormalities whereas in the control group, no ECG abnormalities were reported^34^. In another research, minor and major ECG abnormalities were common in all diabetics (29.1%), including patients with no CVD history (24%)^35^. A small study in Sub-Sahara Africa mentioned conduction defects (11.9%) and arrhythmias (16.2%) as the two most common ECG abnormalities among diabetic patients^36^. The high prevalence of these two abnormalities might be attributed to missed ischemic heart daises and/or contractile disorders as consequence of disturbed Ca^2+^ handing triggered by T2DM, a suggested mechanism of diabetic cardiomyopathy^37^.

Physical activity levels were associated with minor and major abnormalities in this study. Commonly, physical activity is advised for the prevention of CVD. However, some electrical and structural changes occur in the heart of athletes secondary to intense exercise including sinus bradycardia, early repolarization, prolongation of QT interval, and first-degree atrioventricular block^38,39^. In our study, smoking was not significantly associated with major and minor ECG abnormalities. A study by Michelle et al. showed that ECG abnormalities are more common in cigarette smokers compared to the healthy individuals^40^. In another investigation, the ECG of 532 smokers recorded in the baseline and after 3 years. Major ECG abnormalities significantly were related to higher pack-years. Almost 43 percent of participants quit smoking after three years^41^. The reason for this discrepancy is not clear but it may be due to the number of pack-years in participants and the duration of being smoker. Further studies, which take these variables into account, will need to be undertaken.

We didn’t find any association between minor and major ECG abnormalities with obesity and hypercholesterolemia. In line with our study, two other studies also denied any association between left ventricular hypertrophy patterns in ECG and obesity^42,43^. However, it is previously identified that obese cases may have ECG changes including, low QRS voltage, left ventricular hypertrophy, and leftward shifts of the P wave QRS and T wave axes^44^. An analysis of 302 women aged 50 to 69 years showed that abdominal obesity and hyperlipidemia had no association with ventricular repolarization^45^.

In spite of our findings, obesity was reported to be associated with the prolongation of PR interval and the widening of QRS^46^. This disparity may be due to the heterogeneity of our data or confounding variables. In addition, there are different types of obesity phenotypes including metabolic healthy and metabolic unhealthy obesity which didn’t separate in this research. Researchers showed that poor metabolic health is more important than BMI and fat mass for the development of CVD^47^. According to current evidence, the metabolic unhealthy obesity phenotype is more predisposed to cardiovascular events^48^. Thus, we suggest future studies to investigate the association of ECG abnormalities with obesity phenotypes.

In another part of this research, the association between specific minor and major ECG abnormalities and the number of CVD risk factors was also analyzed separately. We observed that in men, as the number of CVD risk factors increases, the frequency of the presence of minor isolated Q/QS waves, high R waves (left ventricular), and major abnormalities increase as well. In women, the frequency of minor abnormalities, minor isolated Q/QS waves, minor isolated Q/QS waves, long PR interval, sinus tachycardia, left atrial enlargement, major abnormalities, complete or intermittent RBBB, and nonspecific intraventricular block were associated with the higher number of CVD risk factors. A similar study among 16 415 Hispanic/Latinos reported there was a significant trend for increasing prevalence of minor isolated ST-T abnormalities, left ventricular hypertrophy plus major ST-T abnormality, major Q waves, major isolated ST-T abnormalities, and ventricular conduction defects with the number of CVD risk factors and prevalent CVD in men. In women, minor isolated ST-T abnormalities, minor isolated Q waves, tall R waves, major Q waves, major isolated ST-T abnormalities, left ventricular hypertrophy plus major ST-T abnormality, and major QT prolongation index with the number of CVD risk factors and prevalent CVD^49^.

The key strength of the study is that an in-depth analysis was undertaken to investigate the relationship between CVD risk factors and ECG abnormalities in a large sample size. However, a number of limitations need to be noted regarding the present study. It is a cross-sectional study, so the causal relationship cannot be determined. Also, the individuals over 65 years and/or lower than 35 years were not included in this study. Another limitation is the fact that only one ECG was obtained at the beginning of the study, ECG criteria can be dynamic and could be more useful if multiple ECGs were obtained at various time points.

## Conclusion

This study showed that age and HTN significantly associated with major and minor ECG abnormalities in women, and HTN and DM were associated with major abnormalities in men. Therefore, pursuing therapeutic lifestyle modification to decrease blood pressure and blood glucose are realistic and testable approaches to lower the risk of some prognostically important ECG abnormalities.

## Data Availability

The authors confirm that the data supporting the findings of this study are available from the corresponding author on request.
